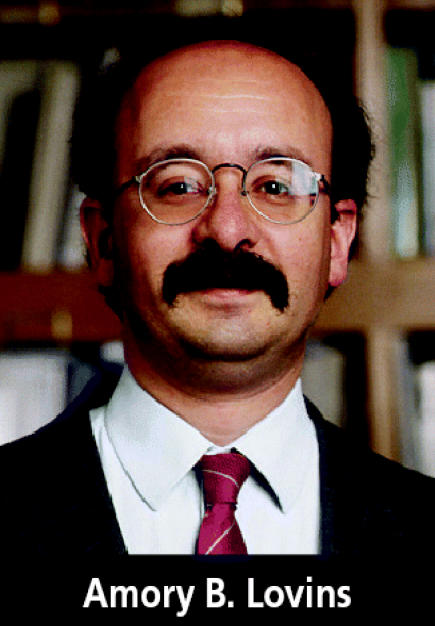# Guest Editorial: Reinventing the Wheels

**DOI:** 10.1289/ehp.113-a218

**Published:** 2005-04

**Authors:** Amory B. Lovins

**Affiliations:** Rocky Mountain Institute, Snowmass, Colorado, E-mail: ablovins@rmi.org

Light-duty vehicles are amazing artifacts, made by the world’s largest industry and fueled by the second-largest. American automakers make a new light vehicle every 2 seconds. Cheaper per kilogram than a fast-food hamburger, those vehicles meet conflicting requirements for performance, emissions, fuel economy, esthetics, and safety with remarkable skill. Yet, a typical light vehicle releases its own weight in carbon dioxide annually, and each day it consumes gasoline made from about a hundred times its own weight in ancient plants (Lovins et al. 2004). Cars and light trucks use 42% of America’s 10,000 gal/second oil habit and account for 58% of projected growth in U.S. oil use to 2025 [Energy Information Administration (EIA) 2004].

At the root of the problem is technology that has improved only incrementally, not radically, since the 1920s. After more than a century of devoted engineering effort, today’s cars use < 1% of their fuel energy to move the driver: only 13% of the fuel energy reaches the wheels, and just 6% accelerates the vehicle, 95% of whose weight is its own, not the driver’s. Two-thirds (or, not counting accessory loads, three-fourths) of the fuel use is caused by the car’s weight (An and Santini 2004), and each unit of fuel saved at the wheels saves an additional seven units of fuel lost en route to the wheels. Obviously, then, the most powerful way to reduce fuel use and emissions of cars is to reduce their mass radically—say, by half.

Automakers have not yet taken this approach very seriously because they assumed reducing mass meant using light metals such as aluminum and magnesium, whose cost is barely justified to save gasoline that in the United States costs less than bottled water, and because they thought lightweighting would compromise safety in collisions with heavier vehicles. Fortunately, these objections are now fading. Both carbon fiber composites and light, high-performance steels now hold promise of equal or better economics and safety with roughly redoubled fuel economy—after it has already doubled by today’s best hybrid-electric powertrains (Holzman 2005; Lovins and Cramer 2004; Lovins et al. 2004). Moreover, even the relatively costly advanced composites may not make cars costlier: the cost of their extra materials can be offset by simpler automaking and smaller power trains (Lovins et al. 2004). Thus an ultralight hybrid-electric vehicle could cost slightly more because it is a hybrid, but not because it is ultralight, and it could repay its extra cost in 3 years, from saved gasoline costing $1.44/gal. Its 69% fuel saving would be equivalent to buying gasoline at just $0.57/gal ($0.15/L) (Lovins et al. 2004). This assumes no further innovation; yet, Toyota’s hybrid power trains, being based largely on software and electronics, have improved more in the past 6 years than U.S. nonhybrid ones have in the past 26 years.

The attractions of superefficient, cost-effective, but uncompromised vehicles can be further increased by public policies that use neither mandates nor fuel taxes (Lovins et al. 2004). Most important are revenue- and size-neutral “feebates”: within each size class, buyers of efficient cars get rebates, paid for by corresponding charges on inefficient cars. This causes producers to add efficiency improvements up to the rebate value; buyers to consider fuel savings over the vehicle’s life, not just the first few years; and both parties to profit. This and other lighthanded policies (smart procurement, “golden carrot,” and “platinum carrot” rewards to reduce the risks and increase the rewards of innovation, low-income scrap-and-replace programs) can speed ultralight hybrids to market by 2010 and give them a 90–100% market share by 2040. In contrast, the U.S. government forecasts (EIA 2004) that without business- or policy-driven innovation, light vehicles will spend the next 20 years getting 1.5 miles/gal more efficient than they were in 1987, and even incrementally improved versions that save a quarter of the fuel and pay back in a year—as proposed by the National Research Council (2001)—will scarcely sell at all.

What will most create this automotive revolution, though, is not public policy but market competition. Toyota already has greater market capitalization than General Motors, Ford, and DaimlerChrysler put together, and their last profitable niche—big sport utility vehicles and pickups—is under frontal assault by Japanese and European firms. Next comes China, which intends to become a major exporter by 2010, has a visionary efficiency- and leapfrog-based energy policy, and is unlikely to export cars as inefficient as most U.S.-made ones, which are gradually becoming illegal to sell under China’s new efficiency standards. How long will it take before you drive home a Wal-Mart-badged superefficient car from Shanghai Automotive? Maybe as little as a decade. The issue for the United States is only whether to import efficient cars to replace foreign oil or to make efficient cars and import neither the oil nor the cars.

Both competitiveness and security concerns reinforce the already-strong economic case for the United States—and others—to stop using oil altogether. Traditionally, this was assumed to require government policies to force something basically uneconomic (or we would already have done it). However, a detailed 2004 analysis (Lovins et al. 2004) found that saving half the oil (at an average cost of $12/barrel in 2000 dollars) and replacing most or all of the rest with advanced biofuels and saved natural gas (at an average cost below $18/barrel) costs less than the private internal cost of buying oil in the world market, even if its externalities were worth nothing. Thus the transition beyond oil can be led by business for profit, and if done right, it can also rejuvenate the economy. Given superefficient vehicles, hydrogen (Lovins 2004) is the most efficient and profitable way to use the saved natural gas, but is not essential to displacing oil.

Other innovations can help, too (Hawken et al. 1999). Too many people driving too many kilometers in too many cars will not work, even if the cars are very clean and efficient: we will simply run out of roads, land, and patience rather than air, oil, and climate. By no longer subsidizing and mandating sprawl, we can already be where we want to be, so there is no need to go elsewhere. We can move only electrons and leave the heavy nuclei at home. We can have real competition at honest prices between all ways of getting around or not needing to, so drivers get what they pay for and pay for what they get. As Alan Durning says, what we need is not better cars but better neighborhoods (Tyson 1998), so we can make cars “an accessory of life rather than its central organizing principle” (Durning 1998).

Physicist Amory Lovins is cofounder and CEO of Rocky Mountain Institute and the chairman of Fiberforge. Published in 29 books and hundreds of papers, his work has been recognized by the “Alternative Nobel,” Onassis, Nissan, Shingo, and Mitchell Prizes, a MacArthur Fellowship, the Happold Medal, nine honorary doctorates, and the Heinz, Lindbergh, World Technology, and “Hero for the Planet” Awards.

## Figures and Tables

**Figure:**